# Cholecalciferol (Vitamin D_3_) Improves Myelination and Recovery after Nerve Injury

**DOI:** 10.1371/journal.pone.0065034

**Published:** 2013-05-31

**Authors:** Jean-Francois Chabas, Delphine Stephan, Tanguy Marqueste, Stephane Garcia, Marie-Noelle Lavaut, Catherine Nguyen, Regis Legre, Michel Khrestchatisky, Patrick Decherchi, Francois Feron

**Affiliations:** 1 Aix Marseille Université, CNRS, NICN UMR 7259, Marseille, France; 2 APHM, Hôpital de la Conception, Services de Chirurgie de la Main, Chirurgie Plastique et Réparatrice des Membres, Marseille, France; 3 Aix Marseille Université, CNRS, ISM UMR 7287, Marseille, France; 4 Aix Marseille Université, Service Hospitalier d'Anatomie et Cytologie Pathologiques Humaines, Assistance Publique - Hôpitaux de Marseille, Marseille, France; 5 Aix Marseille Université, INSERM U1068, Marseille, France; 6 Aix Marseille Université, INSERM, TAGC UMR U1090 Marseille, France; Albert Einstein College of Medicine, United States of America

## Abstract

Previously, we demonstrated i) that ergocalciferol (vitamin D_2_) increases axon diameter and potentiates nerve regeneration in a rat model of transected peripheral nerve and ii) that cholecalciferol (vitamin D3) improves breathing and hyper-reflexia in a rat model of paraplegia. However, before bringing this molecule to the clinic, it was of prime importance i) to assess which form – ergocalciferol versus cholecalciferol – and which dose were the most efficient and ii) to identify the molecular pathways activated by this pleiotropic molecule. The rat left peroneal nerve was cut out on a length of 10 mm and autografted in an inverted position. Animals were treated with either cholecalciferol or ergocalciferol, at the dose of 100 or 500 IU/kg/day, or excipient (Vehicle), and compared to unlesioned rats (Control). Functional recovery of hindlimb was measured weekly, during 12 weeks, using the peroneal functional index. Ventilatory, motor and sensitive responses of the regenerated axons were recorded and histological analysis was performed. In parallel, to identify the genes regulated by vitamin D in dorsal root ganglia and/or Schwann cells, we performed an *in vitro* transcriptome study. We observed that cholecalciferol is more efficient than ergocalciferol and, when delivered at a high dose (500 IU/kg/day), cholecalciferol induces a significant locomotor and electrophysiological recovery. We also demonstrated that cholecalciferol increases i) the number of preserved or newly formed axons in the proximal end, ii) the mean axon diameter in the distal end, and iii) neurite myelination in both distal and proximal ends. Finally, we found a modified expression of several genes involved in axogenesis and myelination, after 24 hours of vitamin supplementation. Our study is the first to demonstrate that vitamin D acts on myelination via the activation of several myelin-associated genes. It paves the way for future randomised controlled clinical trials for peripheral nerve or spinal cord repair.

## Introduction

In a previous study, using a rat model of nerve trauma, we demonstrated that vitamin D_2_ is a potent compound that promoted axon sparing/regeneration and improved physiological maturation [1]. We also observed that vitamin D_2_ supplementation induced an increase in axon diameter, suggesting that myelination was probably enhanced [1]. However, we had no direct evidence that vitamin D is a true myelinating agent.

Vitamin D is a group of seco-steroid hormones, including the fungi-derived form of vitamin D, named vitamin D_2_ or ergocalciferol, and the animal-derived form of vitamin D, named vitamin D_3_ or cholecalciferol. After two separate hydroxylations, performed by two P450 enzymes (25-hydroxylase and 1-alpha-hydroxylase, respectively), both calciferols give rise to the active form (1,25(OH)2D), referred to as calcitriol [2]. Initially, it was thought that liver and kidneys were the only organs responsible for the production of calcitriol. However, it is now clearly established that many tissues, including the brain [3], express vitamin D 1-alpha-hydroxylase. Moreover, vitamin D receptors (VDRs) are widely distributed throughout the brain, in rats [4], [5], [6], [7], [8] as well as in humans [3].

Like other neurosteroids, the genomic action of calcitriol is mediated by a nuclear receptor, the VDR, a member of the steroid/thyroid hormone super-family of transcription regulation factors. After hetero-dimerisation with nuclear receptors of the retinoic X receptor (RXR) family, the VDR and its ligand bind to vitamin D responsive elements (VDRE), located in the promoter regions of hundreds of target genes [9]. For example, a VDRE has been found upstream from genes coding for Brain Derived Neurotrophic Factor (BDNF), Nerve Growth Factor (NGF) and Neurotrophin 3 (NT3) [9]. As a result, vitamin D regulates the expression of NGF [10], [11], [12], NT3 and NT4 [13], and Glial cell line-Derived Neurotrophic Factor (GDNF) [14]. When added to cultured hippocampal cells, calcitriol increases neurite outgrowth [12]. Conversely, when vitamin D is removed from the diet of pregnant rat females, decreased expression of NGF is observed in the brains of neonate [15] and adult offspring [16].

However, very little is known about the role of vitamin D during myelination. It is established that the VDR is present in both oligodendrocytes and Schwann cells. When added to cultured myelinating cells, calcitriol induces an upregulation of the transcripts coding for VDR and NGF [17], [18] but has no effect on the mRNA level of Myelin Basic Protein (MBP) or ProteoLipid Protein (PLP) [17]. In order to better understand the putative role of vitamin D on myelination, we performed a comparative pangenomic transcriptome study, after a 24-hour incubation of dorsal root ganglion cells and/or Schwann cells with calcitriol.

Calciferols are FDA-approved molecules used for preventing rickets or treating psoriasis. Nonetheless, there is currently no indication for neurological disorders or trauma. Therefore, in order to move closer to patients, we devised a pharmacological study based on the weekly delivery of an oral dose (low or high) of either ergocalciferol or cholecalciferol. For the low dose, we maintained our initial choice of 100 IU/kg/day (700 IU/kg/week) that potentiated some functional recovery [1]. For the high dose, we elected the concentration of 500 IU/kg/day (3,500 IU/kg/week) that has proven to be safe in humans. A study looking at graded doses of cholecalciferol, delivered daily to 38 healthy men during 8 weeks, found that the dose of 50,000 IU (the equivalent of a bolus of 500 IU/kg/day for a man weighing 100 kg) was safe, without jeopardizing the phosphocalcic homeostasis [19]. A similar outcome - no hypocalcaemia and no hypercalciuria - was reported by two further studies on escalating doses of cholecalciferol (up to 88,000 IU/day), administered during long periods (up to 6 years) to patients with multiple sclerosis [20], [21]. In order to avoid repeated handling of the animals, we also opted for a weekly administration of calciferol, a choice that is supposed not to interfere with vitamin D efficiency since it takes 2 months to return to baseline level after administration of a single high dose of cholecalciferol [22].

## Materials and Methods

### Animals

Six-day-old (for DRG cultures) and 5-week-old (for Schwann cells) Sprague Dawley (Charles River®, Les Oncins, France) male rats were used for the *in vitro* study and 8-week-old male Sprague Dawley rats, weighting 250–300 g (Charles River®, Les Oncins, France) were used for the pharmacological study. All animals were housed in smooth-bottomed plastic cages at 22°C with a 12-hour light/dark cycle. Food (Purina®, rat chow) and water were available *ad libitum*.

Anaesthesia and surgical procedures were performed according to the French law on animal welfare and the Animal Care Committees of Aix-Marseille University and the? CNRS (*Centre National de la Recherche Scientifique*) approved our protocols. Furthermore, experiments were performed following the recommendations provided in the *Guide for Care and Use of Laboratory Animals* (U.S. Department of Health and Human Services, National Institutes of Health) and in accordance with the European Community council directive of 24 November 1986 (86/609/EEC).

### Experimental Design

In a first set of experiments, the rats (n = 36) were randomised into six groups. A Control (C) group (n = 6) included animals on which no surgery was performed. Animals were deeply anaesthetised (Pentobarbital Sodique® Sanofi Santé Animale, 60 mg/kg). Surgical procedures were performed aseptically under binoculars. The peroneal nerve from the left limb was dissected free from the surrounding tissues on a length of 3–4 cm and a 1 cm segment was removed. The nerve segment was immediately replaced in inverted position, and sutured at the two free nerve stumps by three epineurial stitches (Ethilon® 10–0, Ethicon Inc., Johnson & Johnson Company, Auneau, France). Muscles and skin were stitched (Vicryl® 3–0, Ethicon Inc., Johnson & Johnson Company, Auneau, France).

Immediately after lesioning, rats were orally fed weekly with a 500 μl bolus of either ergocalciferol, at the dose of 100 IU/kg/day (D2–100 group, n = 6) or 500 IU/kg/day (D2–500 group, n = 6), or cholecalciferol, at the dose of 100 IU/kg/day (D3–100 group, n = 6) or 500 IU/kg/day (D3–500 group, n = 6), or the excipient (triglycerides) (Vehicle group, n = 6). Rats were weighted every week and the delivered vitamin D doses were adjusted accordingly. For example, a 300 g rat from the D-100 groups received a weekly dose of 210 IU. All compounds were purchased from Crinex Laboratories (Montrouge, France). In a second set of experiments, we increased the statistical power of our study by replicating experiments with 18 rats randomised into 3 groups, as follows: Control group (n = 6); Vehicle group (n = 6) and D3–500 group (n = 6).

### Serumal Vitamin D Assessment

At 3 months post-surgery and one week after the last vitamin D delivery, blood was collected from the Vehicle, D2–500 and D3–500 groups (n = 12 for Vehicle and D3–500 groups; n = 6 for D2–500 group). Serumal calcidiol (25(OH)D2+25(OH)D3) levels were quantified with a standard commercial radioimmuno-assay (La Timone Hospital, Marseille, France).

### Functional Assessment of Hind Limb Recovery

The peroneal functional index (PFI) indicating the functional alteration of the experimental nerve when compared with the opposite side was calculated by the method of Bain et al. [23] with the following formula: PFI = 174.9[(ePL-nPL)/nPL]+80.3[(eTS-nTS)/nTS]-13.4. The PFI recovery rate was defined with a score from −100 to −13.4, where −13.4 represents normal function and −100 a total absence of contraction. Hindlimb paws were marked with black ink and footprints were recorded each week on paper track, copied in a high-resolution scanner, and digitalised images were analysed. Data concerning each animal were individually identified. The parameters measured for both normal (n) and operated (e) feet were footprint length (PL, or longitudinal distance between the tip of the longest toe and the heel) and total toe spread (TS, or cross-sectional distance between the first and fifth toes). Footprints were obtained and analysed on a weekly basis, from the second to the eleventh week after surgery, by an investigator blinded to the treatment groups. All animals had been conditioned to walk homogeneously into the recording apparatus three times per day over five days during the week before surgery.

### Electrophysiological Recordings

Twelve weeks after surgery, rats were re-anaesthetised by an intra-peritoneal injection of solution containing sodium pentobarbital (Pentobarbital Sodique®, Sanofi Santé Animale, 60 mg.kg^−1^). A tracheotomy was performed and rats were artificially ventilated (Harvard® volumetric pump: rate 40–60 min^−1^, tidal volume 2–4 ml, Southmatick, MA, USA). The left peroneal nerve was dissected free from surrounding tissues on a length of 3–4 cm.

#### Maximum relaxation rate

The twitch contraction of the *tibialis anterior* by nerve stimulation was induced with a neurostimulator (Grass S88K®, Grass Technologies, Astro-med Inc., Rhode Island, USA) rectangular delivering, through an isolation unit, a single shock (duration: 0.1 ms, frequency 0.5 Hz) and measured with a strength gauge (Micromanometer 7001®, Ugo Basile Srl., Biological Research Apparatus, Comerio VA, Italy). Twitches were analysed in terms of peak amplitude (A) and maximum relaxation rate (MRR), defined as the slope of a tangent drawn to the steepest portion of the relaxation curve. MRR was normalised to the total twitch amplitude (MRR/A = mean relaxation rate constant, ms^−1^), as suggested by Esau et al. [24] who showed that MRR values are linearly related to A. Twitches were recorded with a Biopac MP150® system (sampled at 2000 Hz, low-pass filtered at 150 Hz) and analysed with the AcqKnowledge® 3.7.3 software.

#### Tetanus threshold

Muscle stimulation was induced by a pair of steel stimulating electrodes (inter-electrode distance: 4–5 mm) placed on the surface of the *tibialis anterior* muscle. Contractions were produced by the neurostimulator (Grass S88K®) delivering trains of rectangular pulses. After determining a threshold able to elicit a twitch, pulse train intensity was set to a supramaximal level. Tetanic threshold was obtained by increasing frequency by 5 Hz steps. The voltage was 20% higher than the voltage evoking a maximal twitch. The duration of stimulus trains was 500 ms, and trains were repeated each second to produce a series of contractions. Pulse duration was 2 ms and five single stimulations were delivered during each 500 ms train (10 Hz).

#### Ventilatory response

According to one of our previous studies [25], changes in ventilation were recorded after *tibialis anterior* stimulation. The experiments were performed after regional circulatory occlusion which isolated and maintained the neural drive and abolished humoural communication. Repetitive muscle stimulation induces fatigue, which activates the muscle metabosensitive afferent fibres projecting to the bulbar respiratory centre and subsequently increases ventilation.

To elicit electrically-induced muscle fatigue (EIF), rhythmic muscle contractions were produced by the neurostimulator (Grass® S88, Quincy, MA) which delivered pulse trains to the muscle surface electrode (pulse duration: 0.1 ms; frequency: 10 Hz, i.e., 5 shocks in each 500 ms train; duty cycle: 500/1500 ms, voltage range: 5 to 8 volts). The voltage was supramaximal, i.e. 20% higher than that used to elicit a maximal twitch. Fatigue was assessed from the decay of force throughout the 3-min EIF period. The strength of muscle contraction (the decay of force) was measured from the beginning to the end of this 3-min muscle electrical stimulation. We chose to stimulate the muscle directly because we previously showed that muscle low frequency stimulation is a strong activator of metabosensitive afferent fibres [26]. Ventilatory activity was recorded using a thermocouple inserted into the tracheal canula. Measurement was performed two minutes before EIF (rest condition) and 5 minutes after, and expressed in cycles/min. Changes in ventilatory activity after EIF was expressed in percent [Δcycle/min (%)] of the mean cycles recorded two minutes before muscle stimulation.

#### Afferent activity

The proximal portion of the peroneal nerve was cut. In order to record the afferent activity from the *tibialis anterior* muscle, a few millimetres of the epineurial tissue were removed from the free end of the distal nerve using an operating microscope (x40, MZ75®, Leica, Heerbrugg, Switzerland). Then, the distal nerve was positioned on a monopolar tungsten electrode and immersed in paraffin oil. Nerve activity was recorded in reference to a nearby ground electrode implanted in a close muscle, amplified (50 to 100 K) and filtered (30 Hz to 10 kHz) by a differential amplifier (P2MP® SARL, Marseille, France). The afferent discharge was recorded (Biopac MP150® and AcqKnowledge® software, BIOPAC Systems, Inc., Goleta, USA) and fed into pulse window discriminators (P2MP® SARL, Marseille, France), which simultaneously analysed afferent populations. The output of these discriminators provided noise-free tracings (discriminated units) that were counted by a data analysis system (Biopac AcqKnowledge® software BIOPAC Systems, Inc., Goleta, USA) at 1 s intervals (in Hz) and then displayed on a computer. The discriminated units were counted and recorded on separate tracings.

As previously described [1], [2], we recorded the response of muscle afferents after 1) a 3 min low frequency (10 Hz) electrical stimulation of the *tibialis anterior* muscle [simulated fatigue - Electrically-Induced Fatigue (EIF)], 2) an intra-muscular injection of capsaicin solution (655 μM in 50 µL of saline). The discharge rate of nerve afferents was averaged for a 1-min period preceding EIF or capsaicin injection (baseline discharge), and its maximal change was measured following activation. The increase in average afferent discharge rate after EIF or capsaicin injection was expressed as a percentage of average afferent discharge rate before activation. A 20-minute recovery period was allowed between EIF and capsaicin injection.

### Muscular Atrophy Measurement

After the electrophysiological study, rats were sacrificed by an intra-arterial overdose (1 ml) of sodium pentobarbital solution (Pentobarbital Sodique®^,^ Sanofi Santé Animale, 60 mg.ml^−1^). The left *tibialis anterior* muscle was harvested and immediately weighted on a precision scale (Navigator™, N30330 model, OHAUS Corp., New Jersey, USA). Comparisons of muscle mass atrophy were performed using a muscle weight/body weight ratio.

### Histology and Microscopy

Histology assessments were performed with the three most interesting groups: Control, Vehicle and D3–500. For axon number counting, peroneal nerves (n = 6 per group) were harvested, washed in phosphate-buffered saline (PBS), immersed in a 4% paraformaldehyde-containing PBS solution during 24 hours and sectioned in three parts (proximal end, middle of the segment and distal end) before being immunostained with an anti-neurofilament antibody. For the Control group, a unique section, located at the middle of the virtually-sectioned segment, was collected. In each group, the samples were included in paraffin. After embedding, sections (5 μm) were cut on a microtome (RM2155, Leica^®^, Solms, Germany) and collected on SuperFrost Plus® slides (Gerhard Menzel-Glaser, GmbH, Germany). Then sections were immunostained with a mouse monoclonal antibody to the light chain of the neurofilament protein (NF-L 70 kDa, Dako MO762, dilution: 1/100) using a robot (Benchmark® XT, Ventana Medical Systems, Inc., Arizona, USA). After washing, an appropriate biotinylated-conjugated secondary antibody was applied to the sections. The final staining step was performed using diaminobenzidine (Ventana® iVIEW DAB 760 091, Ventana Medical Systems, Inc., Arizona, USA).

For myelination assessment, peroneal nerves (n = 6 per group) were harvested free from surrounding tissues, washed in PBS (Gibco®, Life Technologies Corp., Saint Aubin, France) and immersed in a 4% glutaraldehyde-containing PBS solution during 24 hours. Samples were stained with *p-*phenylenediamine (PPD). After inclusion, semithin sections (0.8 µm) were cut using an ultramicrotome (Ultracut® R, Leica, Solms, Germany) and collected on SuperFrost Plus® slides. After being dried for 12 hours on a hot plate, sections were stained with a *p-*phenylenediamine-ethanol (70°) solution, washed in distilled water, dried for 5 hours on a hot plate and mounted with glycerol-containing medium (Glycergel®, DakoCytomation, Glostrup, Denmark).

The slides were examined using an optical microscope (Eclipse® E800, Nikon, Champigny-sur-Marne, France) that was associated with a high-resolution colour digital camera (DXM 1200, Nikon). The slides were digitised and analysed with the Histolab software (Alphelys®, Plaisir, France). The following parameters were measured: axon number and axonal area. The counting was performed by a robot and therefore observer-associated biases were avoided. To assess G-ratio (i.e. the ratio between the diameter of the axon and the outer diameter of the myelinated fibre), slides were coded, 5 regions of interest in each section were randomly chosen and data analysis was performed blindly.

### Cultures of Schwann Cells and Dorsal Root Ganglia

Nerves from three rats were collected and the connective tissue surrounding nerve bundles was carefully discarded. Nerves were then cut into small pieces with a McIlwain chopper and enzymatically dissociated during 10 minutes with a solution of tryspin-EDTA (0.25% Tryspin, 1 mM EDTA, Invitrogen^®^, Life Technologies Corp., Saint Aubin, France) diluted in Hank's Balanced Salt Solution (HBSS) and then mechanically dissociated with a flamed Pasteur pipette. The enzyme activity was blocked with 9 ml of DMEM/Ham-F12 supplemented with foetal calf serum (10%) (DH/FCS), penicillin (50 U.ml^−1^) and streptomycin (50 μg.ml^−1^) (Invitrogen^®^), the cell suspension was centrifuged for 5 min at 300 g and the cell pellet was resuspended in DH/FCS before being plated onto poly-L-lysine-coated (2 μg.cm^−2^) flasks. When the required number of cells was obtained, Schwann cells were detached using trypsin (0.25%), centrifuged and re-seeded, at the density of 5,000 cells per cm^2^ in DMEM/Ham-F12 supplemented with insulin, transferrin and selenium (ITS, Invitrogen^®^) and TGFα (25 ng.ml^−1^, Sigma-Aldrich Co., Missouri, USA).

Thoracolumbar dorsal root ganglia (DRG) were removed from young (Postnatal day 6) Sprague Dawley rats, anaesthetised with a lethal dose of inhaled isoflurane. Ganglia were transferred into Dulbecco’s modified Eagle medium (DMEM, Invitrogen^®^) supplemented with penicillin and streptomycin (Invitrogen^®^). The connective tissue was removed and ganglia from 10 rats were collected and seeded in a 6-well plate, precoated with poly-D-lysine (1 mg.ml^−1^, Sigma-Aldrich Co.) and laminin (10 μg.ml^−1^, Sigma-Aldrich Co.,) and cultured in DMEM/Ham-F12 supplemented with serum (10%), penicillin, streptomycin, NGF (50 ng.ml^−1^, Alomone Labs^®^, Jerusalem, Israel). Two days later, the culture medium was removed and replaced by serum-free Neurobasal medium (NB, Invitrogen^®^) supplemented with penicillin/streptomycin (40,000 IU/l, Invitrogen^®^) and B-27 supplement (Invitrogen^®^). Neurons and Schwann cells grew out from the whole ganglia and the culture was maintained as a mixed culture of the two cell types.

### Transcriptome Study

Calcitriol (1,25(OH)D3) (#D1530, Sigma-Aldrich Co.) was added at the concentration of 500 nM, during 24 hours, to serum-free DMEM/Ham-F12 supplemented with insulin, transferrin and selenium. Eight 25-cm^2^ flasks, containing either Schwann cells (n = 4), treated (n = 2) or not (n = 2) with calcitriol or a mixed culture of DRG and Schwann cells (n = 4), treated (n = 2) or not (n = 2) with calcitriol, were used. At the end of the incubation period, cultures of Schwann cells and cultures of DRGs and Schwann cells were trypsinised and centrifuged before being treated with RNeasy lipid minikit (#74804, Qiagen, Life Technologies Corp. California, USA). Unwanted genomic DNA was removed using DNase set kit (#79254, Qiagen). Purified total RNAs, from three pooled replicate cultures, were kept at −80°C and processed for hybridisation on genome-wide DNA microarrays within one month. All RNAs were checked for integrity using the 2100 BioAnalyzer (Agilent Technologies^®^, California, USA) and quantified using a ND-1000 spectrophotometer (NanoDrop, Thermo Fisher Scientific Inc., Massachusetts, USA). Cyanine-3-labeled cRNA was generated from 0.3 mg of RNA using the One-Color Low RNA Input Linear Amplification kit (Agilent Technologies) according to the manufacturer’s instructions, followed by purification on RNeasy column (Qiagen). All amplified cRNAs were checked for dye incorporation, cRNA yield and amplification profile. Only those fitting all quality criteria were fragmented for further hybridisation onto microarrays. Samples were then carefully hybridised onto Agilent Whole Rat Genome (4644K) Oligo Microarrays (G4131F). Microarrays were scanned using an Agilent DNA microarray scanner G2505B. Data are available on the ArrayExpress database (accession number E-MEXP-3491).

### Microarray Data Analysis

Individual microarray quality was evaluated based on QC report, pair-wise MA-plots, and box plots. Intra-array normalisation of raw signals from the 8 microarrays (corresponding to the four above-mentioned conditions in duplicate) was performed using Feature Extraction software 9.1.3.1 (Agilent Technologies^®^). Microarray normalised data were further exported into the Limma package, for inter-array normalisation using the quantile method. Statistical analysis was performed using the TIGR MeV (MultiExperiment Viewer) v4.4 software (http://www.tm4.org/mev.html) and the GeneANOVA program. Multi-way ANOVA model was implemented: first, to identify differentially regulated genes when accounting for the multiple sources of variation in the microarray experiment; second, to evaluate the effect of the main variable, the addition of calcitriol during 24 hours. Multiple test correction was further carried out using the false discovery rate (FDR) method. Cluster and Tree View softwares were used for unsupervised hierarchical clustering.

### Overview of Functional Patterns Altered by Vitamin D Supplementation

As a primary analysis, genes identified to be differentially-expressed were analysed for significant gene ontology clusters using DAVID Bioinformatics Resources (http://david.abcc.ncifcrf.gov/). Gene functional classification was used to rank gene ontology clusters by statistical over-representation of individual genes in specific categories relative to all genes in the same category on the filtered list [27].

As a secondary analysis, biological interpretation of the data from the DNA microarrays was performed using Ingenuity Systems Pathway Analysis (http://www.ingenuity.com/). This database builds networks on candidate genes/proteins and putatively associated genes/proteins according to the data collected in previous publications.

### Quantitative PCR Validation of Under- and Over-expressed Genes

The samples used for the microarray experiment were reverse transcribed. Synthesis of cDNA was performed with oligo dT, RNase Out and M-MLV RT enzyme (Invitrogen^®^). Four genes (*Igf1*, *Prx*, *Spp1* and *Tspan2*) involved in axogenesis and myelination and one housekeeping gene (*Gapdh*) were selected for validation using the quantitative PCR technique. Before selecting *Gapdh* as a housekeeping gene, we checked that its expression remained steady in the gene array database. The PCR was performed using the TaqMan Gene Expression Master Mix (Applied Biosystems^®^ #4369016, Life Technologies Corp., California, USA) with the following conditions: 40 cycles, 15 s at 95°C and 1 min at 60°C. Probes were purchased from Applied Biosystems^®^. The reference numbers for each gene were: Rn01775763 (*Gapdh*); Rn00710306_m1* (*Igf1*); Rn00576815_m1* (*Prx*); Rn00563571_m1* (*Spp1*) and Rn00574907_m1* (*Tspan2*).

### Statistical Analysis

Data processing was performed using a software program (Instat® 3.0, GraphPad software, San Diego, California, USA). Data were expressed as mean ± SEM and were compared by ANOVA tests. Post-hoc group comparisons were performed with Student Newman Keuls multiple post-test comparisons. Results were considered statistically significant, highly significant or very highly significant if the p-value fell below 0.05, 0.01 and 0.001, respectively.

## Results

### A Close to Control Locomotor Recovery is Observed with Cholecalciferol at High Dose

**Figure 1:**
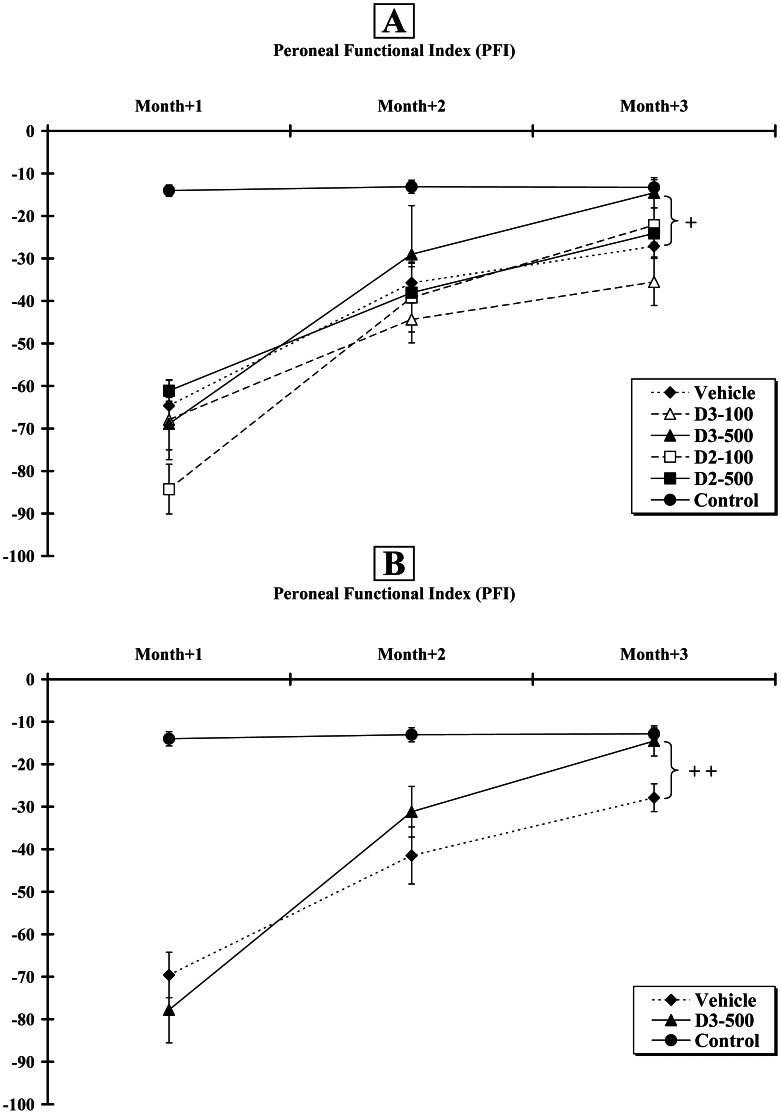
Assessment of Peroneal Functional Index (PFI) at Month+1, +2 and +3 post-surgery. **A.** All animals (n = 6 per group) improved their hindlimb locomotion during the 12 weeks of experiment. **B.** A similar pattern was observed when 12 animals were included in the Control, Vehicle and D3–500 groups. Crosses (+) indicate that the response was significantly increased, when compared to the Vehicle group (+ p<0.05;++p<0.01).

Two-way ANOVA analysis indicated both a group effect [F_5,104_ = 15.8; p<0.001], a time effect [F_2,104_ = 65.0; p<0.001] and interaction between these parameters [F_10,104_ = 3.7; p<0.001]. At Month+2, the scores for the Control, Vehicle, D2–100, D2–500, D3–100 and D3–500 groups were −13.11±1.53; −35.72±3.79; −39.20±8.09; −38.06±7.20; −44.36±5.49; −29.01±11.47, respectively. No difference was found between the operated groups and remained significantly lower than control (p<0.001). At Month+3, the values reached by the same groups were −13.25±1.83; −27.13±2.53, −22.14±4.05, −24.09±2.96, −35.51±5.54 and −14.52±3.54, respectively. When compared to the Vehicle group, only the mean improvement for the D3–500 animals was statistically significant (p<0.05) (**Figure 1A**).

In order to increase the statistical power of our study, we analysed a second set of 6 rats for the Control, Vehicle and D3–500 groups. This replication study for the three groups allowed us to measure more accurately locomotor recovery but also all physiological and histological parameters (*see below*). **Figure 1B** summarises the recovery patterns when the two sets of animals were combined. Two-way ANOVA analysis also indicated both a group effect [F_2,101_ = 44.1; p<0.001], a time effect [F_2,101_ = 45.5; p<0.001] and interaction between these parameters [F_4,101_ = 12.9; p<0.001]. At Month+2, the scores for the Control, Vehicle and D3–500 groups were −13.05±1.65; −41.47±6.70 and −31.17±5.95, respectively. At Month+3, the scores were −12.82±1.37; −27.88±3.27 and −14.52±3.54, respectively. When compared to the Vehicle group, a statistically significant locomotor improvement (p<0.01) was only observed at Month+3.

### Vitamin D Induces a Fast-to-slow Fibre Type Transition of the Tibialis Anterior Muscle

**Figure 2:**
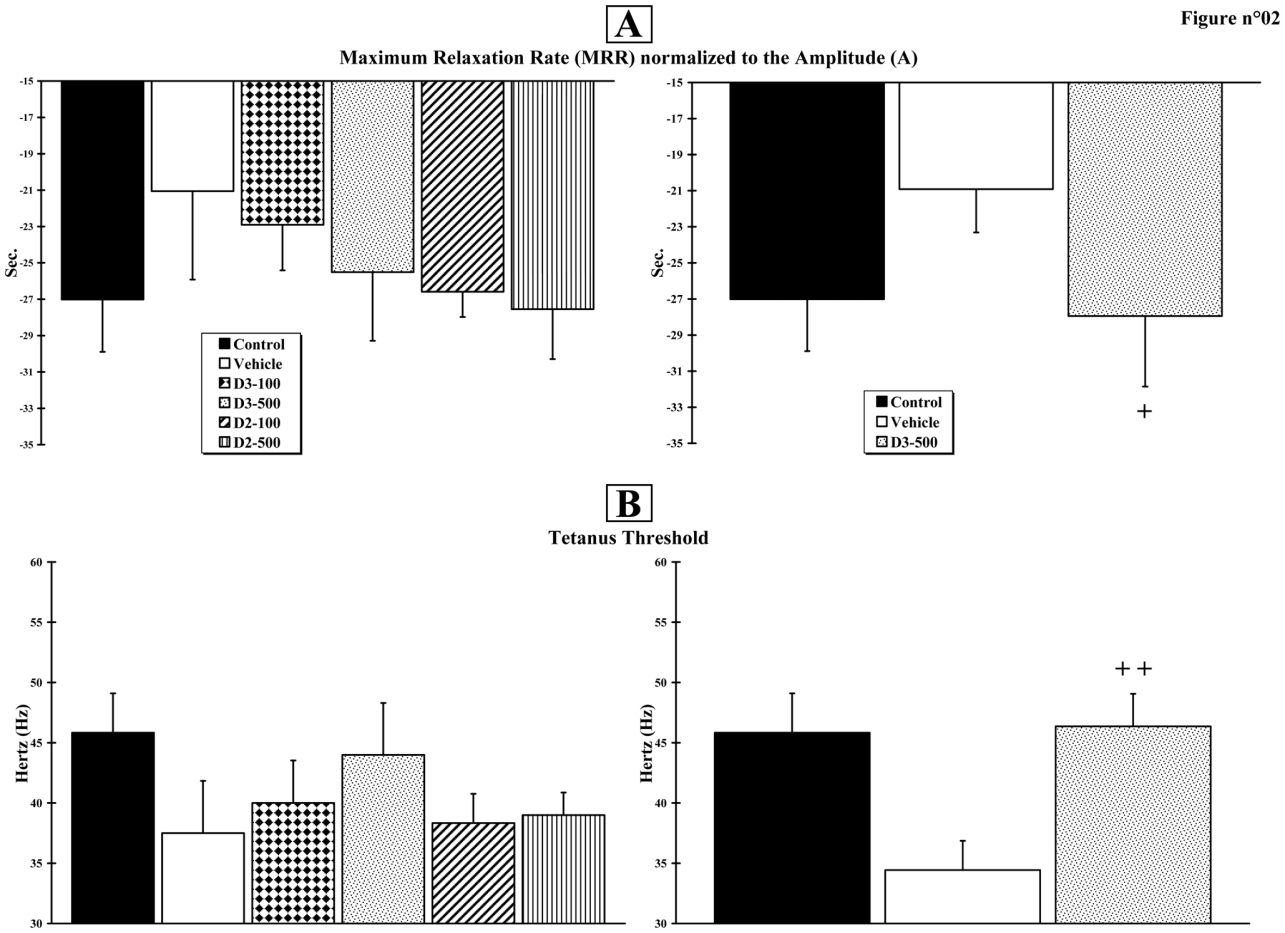
Muscle mechanical properties. Muscle contractions were obtained using peroneal nerve electrical stimulation. **A.** Twitches were analysed in terms of peak Amplitude (A) and Maximum Relaxation Rate (MRR), defined as the slope of a tangent drawn to the steepest portion of the relaxation curve. **B.** Electrical stimulation frequencies were used to reach the tetanus threshold. The experiment was first assessed in the 6 initial groups (n = 6 per group; right histograms) and then in the 3 final groups (n = 12 per group; left histograms). Crosses (+) indicate significant changes when compared to the Vehicle group (+ p<0.05;++p<0.01).

At Month+3, measurement of maximum relaxation rate (MRR), defined as the slope of a tangent drawn to the steepest portion of the relaxation curve and normalised to the total twitch amplitude (A) (MRR/A = mean relaxation rate constant, s^−1^), indicates a trend towards an improvement in all vitamin D-treated groups (**Figure 2A**, left). The values for the Control, Vehicle, D2–100, D2–500, D3–100 and D3–500 groups were −27.01±2.88 s^−1^; −21.05±4.86 s^−1^; −26.58±1.39 s^−1^; −27.54±2.74 s^−1^; −22.90±2.51 s^−1^; −25.50±3.77 s^−1^, respectively. No statistically significant difference was observed. The power of our study was then increased with a second set of 6 rats for the Control, Vehicle and D3–500 groups. **Figure 2A** (right) summarises the recovery patterns when 12 animals were included in each group. The scores for the Control, Vehicle and D3–500 groups were −27.01±2.88 s^−1^; −20.91±2.40 s^−1^ and −27.94±3.90 s^−1^, respectively. The difference between the Vehicle and D3–500 groups reached statistical significance (p<0.05).

Measurement of tetanus threshold obtained by increasing the muscle stimulation frequency indicates a trend towards an improvement in all vitamin D-treated groups (**Figure 2B**, left). The values for the Control, Vehicle, D2–100, D2–500, D3–100 and D3–500 groups were 45.83±3.27 Hz; 37.50±4.33 Hz; 38.33±2.42 Hz; 39.00±1.87 Hz; 40.00±3.53 Hz; 44.00±4.30 Hz, respectively. However, no significant difference was observed.

**Figure 2B** (right) summarises the recovery patterns when 12 animals were included in the final three groups. The scores for the Control, Vehicle and D3–500 groups were 45.83±3.27 Hz; 34.44±2.42 Hz and 46.36±2.70 Hz, respectively. The difference between Vehicle and D3–500 groups was significant (p<0.01).

### Nerve Afferent Responses to Fatigue or Chemicals and Ventilatory Reflex are Improved by Calciferols at High Dose

Air flow movements which represent the ventilatory activity were recorded through the canula inserted into the trachea after EIF activation of the muscle afferent fibres projecting to the bulbar respiratory centre. Changes in ventilatory activity after EIF was expressed in percent [Δcycles/min (%)] of the mean cycles recorded two minutes before muscle stimulation. The afferent units activated by EIF and capsaicin injection displayed a low frequency spontaneous baseline activity (3–10 Hz). An increase in basic tonic activity was observed during the first 20 seconds post-stimulation. Then, the response plateaued for approximately 3 minutes. With this in mind, we compared the experimental groups by measuring changes in basal discharge rate, expressed as ?F*_impulses_*.s^−1^ (% of basal activity), as reported before [28], [29]. The percentages reported in the following paragraphs represent the mean of ?F*_impulses_*.s^−1^ (% of basal activity) in each experimental group.

#### Ventilatory responses to Electrically-Induced Fatigue (EIF)

**Figure 3:**
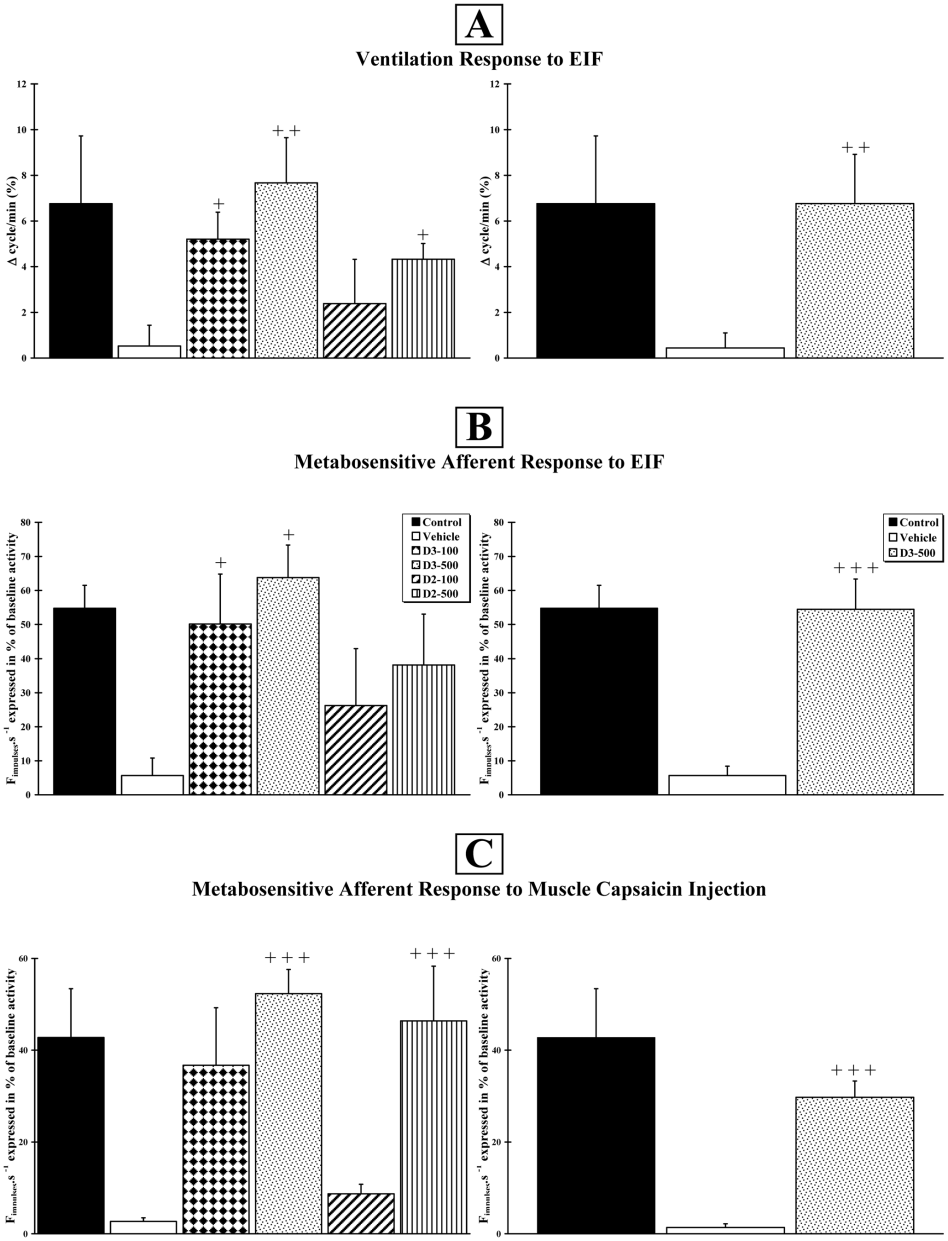
Vitamin D improves responses to muscle electrically-induced fatigue or to a chemical agent. Ventilatory response of the animals after muscle stimulation (**A**) and response of metabosensitive afferent fibre activity after active muscle electrical stimulation (**B**) or intramuscular capsaicin injection (**C**). The experiment was first assessed in the 6 initial groups (n = 6 per group) (right histograms) and then in the 3 final groups (n = 12 per group) (left histograms). Crosses (+) indicate that the response was significantly increased, when compared to the Vehicle group (+ p<0.05;++p<0.01;+++p<0.001).

At Month+3, the scores for the Control, Vehicle, D2–100, D2–500, D3–100 and D3–500 groups were 6.76±2.97%; 0.52±0.92%; 2.39±1.93%; 4.33±0.69%; 5.21±1.18%; 7.67±1.78%, respectively (**Figure 3A**, left). When compared to the Vehicle group, using a one-way ANOVA analysis (F_5,28_ = 2.3; p<0.05), the mean improvement of the D2–500 (p<0.05), D3–100 (p<0.05) and D3–500 (p<0.01) animals were statistically significant. The statistical power of our study was then increased with a second set of 6 rats for the Control, Vehicle and D3–500 groups. The scores for the Control, Vehicle and D3–500 groups were 6.76±2.97%; 0.44±0.66% and 6.77±2.15%, respectively (**Figure 3A**, right). The difference between the Vehicle and D3–500 groups was highly significant (p<0.01) after an ANOVA test (F_2,24_ = 4.2; p<0.05).

#### Responses to Electrically-Induced Fatigue (EIF)

At Month+3, the scores for the Control, Vehicle, D2–100, D2–500, D3–100 and D3–500 groups were 54.73±6.77%; 5.65±5.18%; 26.24±16.72%; 38.15±14.86%; 50.10±14.69%; 63.75±9.58%, respectively (**Figure 3B**, left). When compared to the Vehicle group, a one-way ANOVA (F_5,26_ = 3.1; p<0.05) indicated a statistically significant mean improvement only for the D3–100 and D3–500 animals (p<0.05). The statistical power of our study was then increased with a second set of 6 rats for the Control, Vehicle and D3–500 groups. The scores for the Control, Vehicle and D3–500 groups were 54.73±6.77%; 2.72±2.77% and 54.44±8.91%, respectively (**Figure 3B**, right). After a one-way ANOVA (F_2,25_ = 18.4; p<0.001) and a post-hoc test, the difference between the Vehicle and D3–500 groups was very highly significant (p<0.001).

#### Responses to capsaicin injection

At Month+3, the scores for the Control, Vehicle, D2–100, D2–500, D3–100 and D3–500 groups were 42.77±10.67%; 2.69±0.80%; 8.71±2.08%; 46.41±11.92%; 36.73±12.55%; 52.34±5.25%, respectively (**Figure 3C**, left). A one-way ANOVA (F_5,29_ = 5.8; p<0.001) indicated a statistically significant mean improvement of the D2–500 and D3–500 animals compared to the Vehicle group (p<0.001). The statistical power of our study was confirmed with a second set of 6 rats for the Control, Vehicle and D3–500 groups. The scores for the Control, Vehicle and D3–500 groups were 42.77±10.67%; 1.36±0.83% and 29.76±3.53%, respectively (**Figure 3C**, right). One-way ANOVA (F_2,25_ = 10.7; p<0.001) indicated a significant difference between the Vehicle and D3–500 groups (p<0.001).

### Muscle Weight

Following leg denervation and reinnervation, the relative weight (muscle/animal weight ratio) of the denervated *tibialis anterior* muscle markedly decreased twelve weeks after surgery. Compared to the Control group (0.177±0.003 g.kg^−1^, 100%), the relative weight of the ipsilateral muscle within the Vehicle (0.080±0.003 g.kg^−1^, 45.4%, p<0.001) and D3–500 (0.079±0.005 g.kg^−1^, 44.80%, p<0.001) groups was significantly lighter.

### Cholecalciferol Increases Axon Numbers, Axon Diameter and Myelination

**Figure 4:**
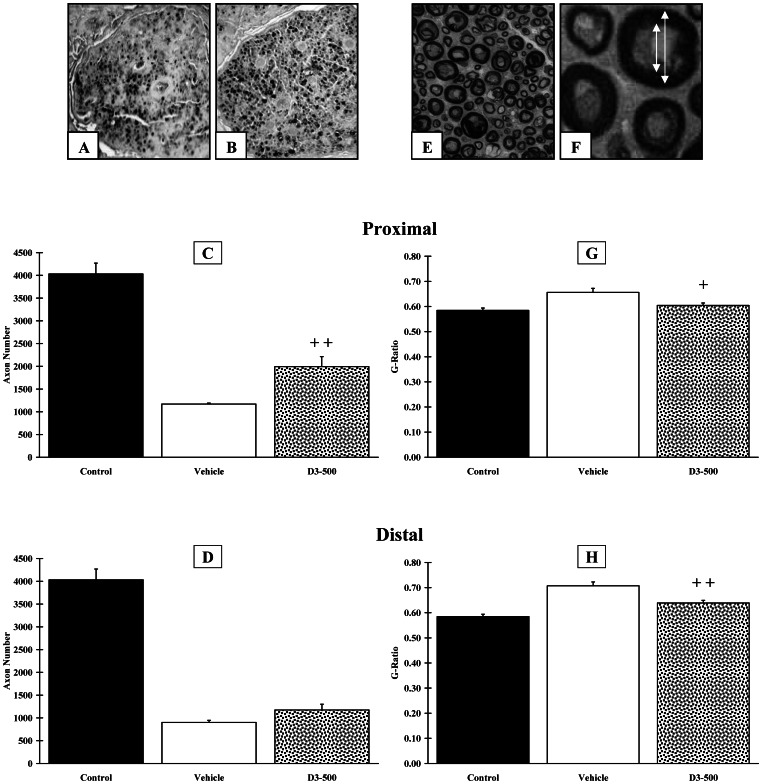
Histological analysis of the peroneal nerve, at 3 months post-surgery. Peroneal nerves from the Control, Vehicle and D3–500 groups were either fixed with paraformaldehyde and included in paraffin (n = 6 per group) for counting axon numbers (**A–D**) or fixed with glutaraldehyde and included in resin (n = 6 per group) for assessing myelination (**E–H**). **A,B.** Representative pictures of nerve sections immunostained with an anti-neurofilament antibody in Vehicle (**A**) and D3–500 (**B**) groups. Quantitative analysis of axon numbers indicated that vitamin D3–500 induced a statistically significant doubling of axons in the proximal end (**C**) but not in the distal end (**D**). **E.** Low magnification view of a nerve section stained with p-phenyl-n-diamine (D3–500 group). **F.** High magnification view of a nerve section with arrows indicating how the G-ratio (the ratio between the diameter of the axon and the outer diameter of the myelinated fibre) was calculated (D3–500 group). Quantitative analysis indicates that vitamin D3–500 triggered myelination in the proximal (**G**) and the distal (**H**) ends of the nerve. Crosses (+) indicate significant changes when compared to the Vehicle group (+ p<0.05;++p<0.01). Scale bar:?

Twelve weeks after surgery, the number of axons (**Figure 4A, B**) in the proximal area for the Control, Vehicle and D3–500 groups were 4031±237, 1169±21 and 1994±220, respectively (**Figure 4C**). Animals from the D3–500 group exhibited a significantly (p<0.01) higher number of axons in the proximal area. The number of axons in the distal area for the Control, Vehicle and D3–500 groups was 4031±237, 901±46 and 1174±126, respectively (**Figure 4D**). No difference was found between groups in the distal area.

When compared to the proximal area, a decrease in mean axon diameter and mean axon surface was observed in the distal area for the Vehicle group (1.98±0.20 µm *vs.* 1.67±0.15 µm and 4.22±0.38 µm^2^ *vs.* 2.93±0.40 µm^2^, respectively). Conversely, the mean axon diameter and mean axon surface remained steady for the D3–500 group (2.06±0.16 µm *vs.* 2.23±0.05 µm and 4.35±0.12 µm^2^ *vs.* 4.92±0.27 µm^2^, respectively). The difference between the Vehicle and the D3–500 groups was statistically significant (p<0.01).

Myelination was assessed using the G-ratio, defined as the ratio between axon diameter and myelinated fibre outer diameter (**Figure 4E, F**). The mean value for the Control group was 0.584±0.009. The mean G-ratio for the D3–500 group (0.604±0.24 and 0.639±0.01, in the proximal and distal areas, respectively) was not statistically different from the Control group. Conversely, a significant difference was observed in both the proximal (p<0.05)(**Figure 4G**) and the distal (p<0.01)(**Figure 4H**) areas, when the D3–500 group was compared with the Vehicle group (0.656±0.016 and 0.708±0.015, in the proximal and distal areas, respectively).

### Vitamin D_3_ Regulates the Expression of Genes Involved in Axogenesis and Myelination

The two protocols used in this study allowed us to get, on the one hand, a pure (over 90%) culture of Schwann cells and, on the other hand, a mixed culture of DRG and Schwann cells. With a fold change threshold set up at 1.5, we observed that 89 and 42 genes were over- and under-expressed in co-cultures of DRG and Schwann cells, respectively, while 126 and 23 genes were over- and under-expressed in cultures of Schwann cells, when calcitriol was added to the culture medium. **Table 1** summarises the list of calcitriol-regulated genes that play a role either in axogenesis or myelination. Six genes (*Acsl4, Arhgef19, Il33, Pank1, Relt* and *Spp1*) were found to be upregulated by calcitriol in both types of cultures.

**Table 1:** Selected calcitriol-regulated genes involved in axogenesis and myelination.

DRG/SC Fold Change	Gene Symbol	Gene Name	SC Fold Change
1.6	**Acsl4**	**Acyl-CoA synthetase 4**	1.7
	Adm	Adrenomedullin	2.3
1.55	**Agtr1a**	**Angiotensin II receptor 1a**	1.6
	Aqp1	Aquaporin 1	1.75
*-2.6*	*Cdh2*	*Cadherin 2*	
1.5	Cdw92	Solute carrier family 44, member 1	
*-3.6*	*Chl1*	*Cell adhesion molecule with homology to L1CAM*	
	*Cacna1b*	*Calcium channel, voltage-dependent, Nalpha1B*	*-2.5*
	Clcf1	Cardiotrophin-like cytokine factor 1	3.1
	Crlf1	Cytokine receptor-like factor 1	1.8
	Cxcl12	Chemokine 12	1.9
	Clu	Clusterin	2.3
	Got1	Glutamic-oxaloacetic transaminase 1	2
	Igf1	Insulin-like growth factor 1	2.4
	Klhl24	Kainate receptor-interacting protein for GluR6	1.7
1.8	Kras	Kirsten rat sarcoma viral oncogene homolog	
1.9	Limk1	LIM domain kinase 1	
*-2.9*	*Map1b*	*Microtubule-associated protein 1B*	
	Mapk8ip1	Mitogen-activated protein kinase 8 interact prot 1	1.8
	Metrn	Meteorin	1.8
1.7	Myc	V-myc myelocytomatosis viral oncogene	
*-5.7*	*Mycbp2*	*MYC binding protein 2*	
1.6	Ncam1	Neural cell adhesion molecule 1	
	Ndrg2	NDRG family member 2	1.9
	*Nefm*	*Neurofilament, medium polypeptide*	*-3.9*
1.9	Ppp3cb	Protein phosphatase 3, catalytic subunit, beta	
3.5	Ppp3r1	Protein phosphatase 3, regulatory subunit B alpha	
2	Prx	Periaxin	
*-2.1*	*Rbpj*	*Rec signal binding prot for IgK J region*	
1.6	Rcan1	Regulator of calcineurin 1	
1.7	Rhoa	Ras homolog gene family, member A	
	*Rtn4rl2*	*Nogo receptor-like 3*	*-2.1*
1.6	Ryk	Receptor-like tyrosine kinase	
2.2	**Spp1**	**Osteopontin**	4
1.6	Strn3	Striatin, calmodulin binding protein 3	
	Thbs4	Thrombospondin 4	2.5
1.5	Trim2	Tripartite motif-containing 2	
	Tspan2	Tetraspanin 2	1.8
1.5	Ulk2	Unc-51 like kinase 2	
	Vegfa	Vascular endothelial growth factor A	1.9

Selected list of transcripts, involved in axogenesis and myelination, whose expression is modified after the addition of calcitriol (100 nM), during 24 hours, to cultures of Schwann cells (SC) or co-cultures of DRG and SC. The symbol, the full name and the fold change of each gene are indicated. Under-expressed genes are in italic. Calcitriol-regulated genes in both Schwann cell and DRG/Schwann cell cultures are in bold.

**Figure 5:**
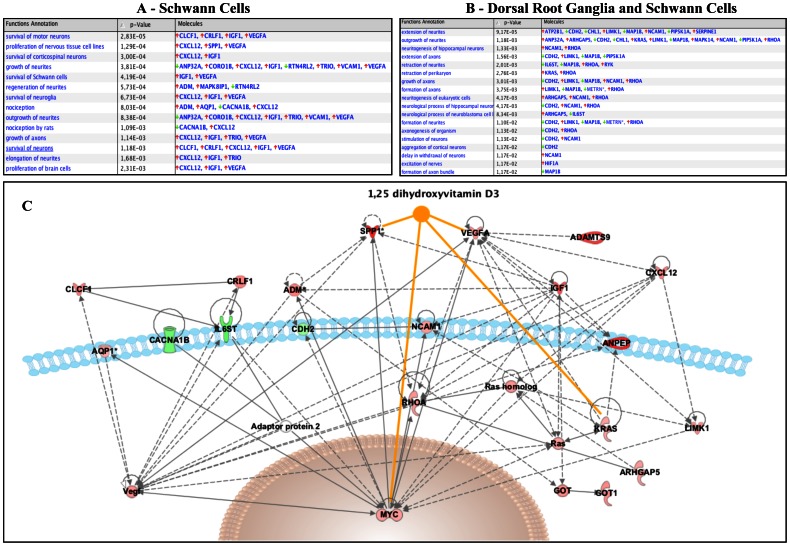
Analysis of the main functions altered by vitamin D supplementation using the Ingenuity Pathway Analysis Tool. **A.** List of functions for the genes involved in “nervous system development and function” whose expression was altered after addition of calcitriol to Schwann cells (**A**) or Schwann cells and dorsal root ganglion cells (**B**). Red arrows indicate an over-expression; green arrows, an under-expression. **C.** Twenty-five nervous system-related genes were used to generate a network representation. The genes shaded red are upregulated and those that are green are downregulated. The intensity of the shading shows to what degree each gene was up- or downregulated. The genes in white colour were not significantly changed in the analysis and can be considered as “missing links”. Orange solid lines represent a known direct interaction between calcitriol and the genes present in the network.

**Figure 5** summarises the functions specifically altered in Schwann cell only cultures (**Figure 5A**) and in DRG/Schwann cell cultures (**Figure 5B**), when data were analysed with the Ingenuity Pathway Analysis Tool. A selection of 25 genes involved in nervous system development and function was used to draw a network representation (**Figure 5C**).

**Figure 6:**
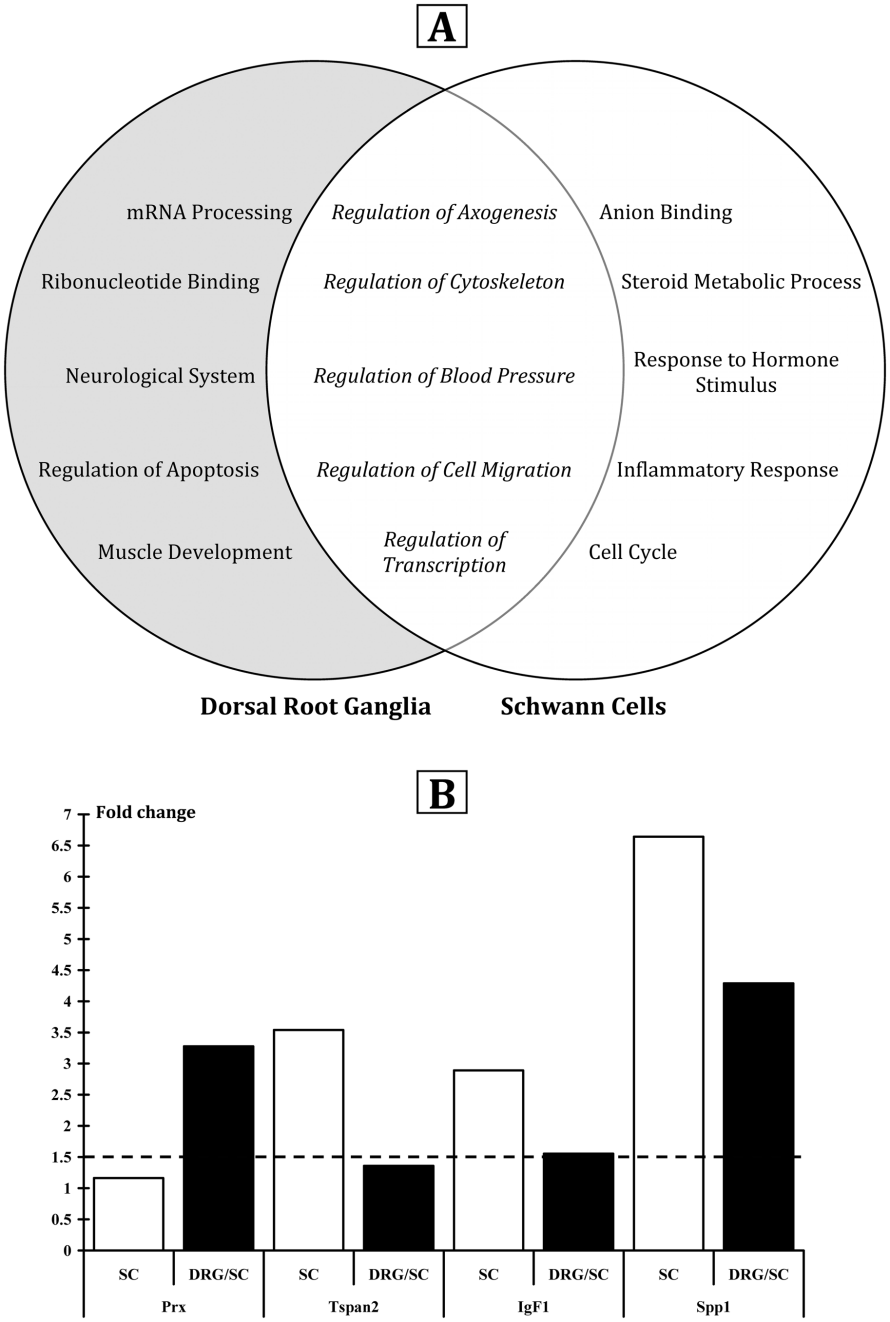
Main metabolic pathways associated to in vitro calcitriol supplementation. **A.** Venn diagram showing the functional pathways affected by the addition of calcitriol in cultures of Schwann cells or in co-cultures of DRG/Schwann cells. Five of the fifteen metabolic calcitriol-regulated pathways are affected in both cell types. **B.** Validation by qPCR of four selected up-regulated genes (*Prx, Tspan2, IgF1, Spp1*) involved in axogenesis and myelination.

Comprehensive microarray data are available on the ArrayExpress database (accession number: E-MEXP-3491). Bioinformatic analysis of altered transcripts with DAVID revealed the main cellular functions modified by vitamin D supplementation. **Figure 6A** summarises the functions specifically altered in DRG/Schwann cell cultures (mRNA processing, ribonucleotide binding, neurological system process, regulation of apoptosis, muscle development) and in Schwann cell only cultures (anion binding, steroid metabolic process, response to hormone stimulus, inflammatory response, cell cycle). Five metabolic pathways were commonly activated in both conditions: *regulation of axogenesis*, *regulation of cytoskeleton*, *regulation of blood pressure*, *regulation of cell migration*, *regulation of transcription*. Within the functional clustering performed with DAVID software, the pathway “regulation of axogenesis” was ranked number one in DRG/SC. In both types of cultures, we also observed genes that are involved in myelination. In order to validate this microarray-generated list, using the same sample material, we performed a quantitative PCR on four selected genes and found that they were upregulated, as expected (**Figure 6B**).

### No Vitamin D-related Hypercalcemia was Observed

Calcium levels were measured and no hypercalcaemia was ever observed. The vitamin D values for the Vehicle, D2–500 and D3–500 groups were 36.3±3.8 ng.ml^−1^ (90.6 nmol/L), 118.1±13.2 ng.ml^−1^ (294.78 nmol/L) and 122±10.5 ng.ml^−1^ (304.51 nmol/L), respectively.

## Discussion

The present study indicates that cholecalciferol (vitamin D_3_) is a more potent neuromodulator than ergocalciferol (vitamin D_2_). It also shows for the first time, in an animal model of nerve trauma, that cholecalciferol, delivered at a high dose, induces a significant locomotor and electrophysiological recovery, with values undistinguishable from control animals, and increases i) the number of preserved or newly formed axons in the proximal end, ii) the mean axon diameter in the distal end and iii) neurite myelination in both the distal and proximal ends. Finally, our study lists the calcitriol-regulated genes that, in cultures of DRG and Schwann cells, are involved in axogenesis and myelination.

### Cholecalciferol vs. Ergocalciferol

There is a long running debate on the form of calciferol that should be delivered to mammals. This is an important issue since many vitamin D supplements that can be purchased over the counter or on the World Wide Web are made with ergocalciferol. Two comparative studies clearly indicate that, in humans, cholecalciferol is more efficient than ergocalciferol [30], [31]. However, a doubt remained about rodents since it has been reported that ergocalciferol is more effective than cholecalciferol in increasing the level of calcidiol (25(OH)D) [32]. Interestingly, the current study sheds new light on this topic: in rats, for nearly all physiological parameters under examination, at the same dose, cholecalciferol is equally or more potent than ergocalciferol.

In our initial study, we demonstrated that ergocalciferol, at the dose of 100 IU/kg/day, induced a modest but significant functional recovery [1]. The absence of improved peroneal functional index, when the animals were treated with vitamin D-100, was replicated. More importantly, we showed that a dramatic improvement could be obtained when ergocalciferol was replaced by cholecalciferol and when a high dose was selected. In our conditions, the serum level of calcidiol raised from 36 ng.ml^−1^ in the Vehicle group to 120 ng.ml^−1^ in D-500-treated animals, without inducing any obvious adverse effect. No evidence for toxicity was observed in a previous study showing that no alteration in plasma phosphate concentrations was found in rats fed with high doses of cholecalciferol (around 400 IU/kg/day), through pregnancy and postnatally during 6 weeks [33]. In dogs, the LD50 (the lethal dose which kills 50% of the animals) for cholecalciferol is over 3,000,000 IU/kg [34]. As reported earlier, it is likely that adipose tissue captures the excess of vitamin D and releases it when fasting conditions occur [35].

Similarly, the oral delivery of cholecalciferol, at doses exceeding 500 IU/kg/day, was found to be safe in humans. No imbalance of the phosphocalcic homeostasis and no hypercalcaemia or hypercalciuria was noticed in humans treated with high doses of cholecalciferol [19], even for long periods [20], [21]. However, hypercalcaemia has been observed in some individuals who ingested more than 40,000 IU/day (for a review, see [36]). It is therefore important to below under this threshold, which corresponds to the ingestion of 500 IU/kg/day for a human weighing 80 kg. One limitation of our study is that the efficiency of an intermediate dose (250 IU/kg/day, for example) has not been tested in our animal model.

### Vitamin D, a Myomodulator?

As demonstrated in our previous study, vitamin D induced a fast-to-slow fibre type transition of the *tibialis anterior* muscle [1]. Furthermore, bioinformatic analysis of our microarray data indicates that the expression of genes involved in muscle development was regulated by calcitriol. However, is it enough to explain the functional recovery observed in the current study? It has been reported that vitamin D directly influences the intracellular accumulation of phosphate in muscle [37] and, when vitamin D is administered to vitamin D-deficient rats, improved muscle anabolism and reduced myofibrillar protein degradation are observed [38]. As a consequence, VDR knockout mice (VDR −/−) display muscular and motor impairments that impinge on their locomotor behaviour [39].

Similar findings have been reported in humans. First, delivery of a vitamin D analogue, for 3 to 6 months, to elderly patients with osteoporosis increased the percentage and area of type-2 (fast-twitch) muscle fibres [40]. Second, a low dose of vitamin D_2_, given during 2 years to 48 vitamin D-deficient elderly hemiplegic women, augmented the relative number and size of type 2 muscle fibres and improved muscle strength [41]. Third, treatment of vitamin D deficiency with vitamin D_3_ and calcium increased lower limb muscle strength, independently of regular physical activity, in institutionalised elderly people [42]. These findings, in combination with studies on athletes, have led experts in the domain to conclude that vitamin D may improve physical performance [43].

However, it must be pointed out that the mean mass of *tibialis anterior* muscle within the vitamin D3–500-treated group was not increased in comparison with the Vehicle group. The functional recovery observed in our study is therefore linked to a qualitative rather than a quantitative change in muscle properties. For example, the improved MRR/A ratio is indicative of improved contractile properties as well as of a change in the percentage of slow and rapid fibres. Furthermore, the physiological recuperation could be the consequence of enhanced neurotransmission, induced by increased axogenesis and myelination.

### Increased Axogenesis and Myelination

Convergent data indicate that vitamin D can be considered as a neuroprotective agent. *In vitro* evidence mainly comes from a number of studies showing that vitamin D treatment i) reduces nitrite production and stimulates γ-glutamyl transpeptidase expression in lipopolysaccharide-stimulated astrocytes [44], [45] and ii) protects mesencephalic dopaminergic neurons from toxins by reducing oxidative stress [46], [47]. Interestingly, these initial results were confirmed by *in vivo* experiments. For example, a vitamin D pre-treatment attenuates the cortical infarction observed after middle cerebral artery ligation [48] and the toxicity induced by 6-hydroxydopamine [49].

In addition to acting as a shield, vitamin D exerts a neurotrophic role. It is now well established that vitamin D stimulates the expression of neurotrophins [10], [11], [13], [14], [18], [50] and increases neurite outgrowth, when added to cultured hippocampal cells [12], [51]. Conversely, when vitamin D is removed from the diet of pregnant rat females, decreased expression of NGF is observed in the brains of neonates [15] and adults [16].

No modification of neurotrophin expression was observed in our transcriptomic study. It can be argued that the cells were cultured in normal conditions and, possibly, vitamin D-regulated neurotrophin gene expression may require a trauma-related inflammation. Furthermore, as indicated by our microarray data, other vitamin D-dependent pathways are probably involved in axon regeneration. **Table 1** lists the 40 gene transcripts, involved in axogenesis and myelination, whose expression has been modified after addition of calcitriol to DRG or Schwann cells. Interestingly, at least 10 of them (*Cdw92, Kras, Metrn, Myc, Ppp3cb, Rtn4rl2, Spp1, Trim2, Tspan2, Vegfa*) include a Vitamin D responsive element (VDRE) in their human promoter regions [9]. Some of these 40 genes are known to play a role in axon growth or guidance. For example, *Igf1* promotes neurite elongation [52] and *Igf1*-deficient mice display a decrease in i) dendritic growth; ii) brain size; iii) axon diameter and iv) conduction velocity [53], [54], [55]. *Metrn* (Meteorin) is a secreted protein that regulates glial cell differentiation and promotes axonal extension [56] whereas the calcineurin/NFAT signalling pathway is a key player in axonal growth and guidance (for a review, see [57]). *Limk1* regulates actin filament assembly at the tip of the growth cone [58], [59] and is critical for calcium signal-induced neurite outgrowth [60] while *Ulk2* triggers filopodia extension and neurite branching [61], [62].

By enhancing axon length and axon diameter, these proteins may be partly responsible for the increased myelination observed in vitamin D-treated animals. However, one of the main findings of our microarray experiment is that calcitriol upregulates genes involved in myelination. *Prx* (Periaxin) is required for the maintenance of peripheral nerve myelin and patients with *Prx* mutations have early-onset autosomal recessive demyelinating Charcot-Marie-Tooth disease (CMT4F) or Déjèrine-Sottas neuropathy (DSN) [63]. *Tspan-2* plays a role in the early stages of oligodendrocyte terminal differentiation into myelin-forming glia and may stabilise the mature sheath [64], [65]. However, to date, nothing is known about its expression in Schwann cell. Finally, *Spp1* (osteopontin) is a well-known vitamin D-regulated cytokine that is associated with ossification, inflammation, chemo-attraction, cancer and hypoxia-induced cell death. More recently, it has been implicated in multiple sclerosis, a demyelinating auto-immune disease [66]. There is also evidence that it acts on axogenesis and myelination. For example, *Spp1* enhances myelin formation *in vitro* [67] and is expressed at high levels during remyelination in an animal model of toxin-induced demyelination [68]. Overall and in the absence of an increased number of axons in the distal part of the nerve, improved myelination is probably the key factor that underlies the dramatic recovery observed in the current study.

### Conclusion

In a previous study, we demonstrated that ergocalciferol potentiates axon regeneration. We show here that cholecalciferol is more efficient than ergocalciferol, inducing a functional recovery reaching control-like values, and, even at a high dose, is non-toxic for the treated rats. We also unveil calcitriol-regulated genes that play a role in axogenesis and myelination. Altogether, our data pave the way for a randomised controlled trial in patients with a peripheral nerve injury.
